# Every Fever Not Merely Due to Antibiotic Deficiency: Chronic Graft-Versus-Host Disease Case Report

**DOI:** 10.7759/cureus.71367

**Published:** 2024-10-13

**Authors:** Nava R Sharma, Madalasa Pokhrel, Prakriti Lamichhane, Sumitra Paudel, Marlon E Rivera Boadla, Prabal KC, Barbara Alvarez

**Affiliations:** 1 Internal Medicine, Maimonides Medical Center, Brooklyn, USA; 2 Medicine, Manipal College of Medical Science, Pokhara, NPL; 3 Internal Medicine, Montefiore Medical Center, New Rochelle, USA; 4 Pathology, KIST Medical College, Lalitpur, NPL; 5 Public Health, The University of Southern Mississippi, Hattiesburg, USA; 6 Research Volunteer, Maimonides Medical Center, Brooklyn, USA; 7 Internal Medicine, Rasuwa District Hospital, Kathmandu, NPL; 8 Infectious Disease, Maimonides Medical Center, Brooklyn, USA

**Keywords:** cutaneous chronic graft versus host disease, cutaneous scleroderma, fever of unknown, graft-versus-host disease (gvhd) remains a major complication for patients undergoing hsct. chronic gvhd (cgvhd), gvhd

## Abstract

This case report presents a 53-year-old female patient with a history of acute lymphoblastic leukemia (ALL) who developed chronic graft-versus-host disease (cGVHD) following an allogeneic bone marrow transplant, leading to significant respiratory distress and notable skin findings, including hyperpigmentation and chronic non-healing ulcers. The patient’s clinical course illustrates the diverse manifestations of cGVHD, emphasizing the importance of recognizing that not all febrile episodes in post-transplant patients are attributable solely to infection. A multidisciplinary approach was essential for accurate diagnosis and management, underscoring the need for comprehensive evaluation of symptoms in immunocompromised patients. This case contributes to the understanding of cGVHD's clinical implications and highlights the necessity for ongoing research in its management.

## Introduction

Chronic graft-versus-host disease (cGVHD) is a significant and complex complication that can arise after allogeneic hematopoietic stem cell transplantation (HSCT), particularly in patients with a history of hematologic malignancies likeacute lymphoblastic leukemia (ALL) [1]. As the donor immune cells mount an attack against the recipient's tissues, cGVHD can manifest in various organ systems, with the skin and lungs being commonly affected [2]. The clinical presentation of cGVHD can be insidious and may overlap with other post-transplant complications, making diagnosis challenging.

We present a case of a 53-year-old female patient who developed respiratory distress and skin findings consistent with cGVHD following her treatment for ALL and subsequent allogeneic bone marrow transplant. This case emphasizes the importance of recognizing the diverse manifestations of cGVHD and the necessity for a multidisciplinary approach to optimize patient outcomes following HSCT.

## Case presentation

A 53-year-old female with a past medical history of pre-B ALL treated with chemotherapy, radiotherapy, and an allogeneic bone marrow transplant (BMT) eight years back presented to the emergency department (ED) with a chief complaint of one week of worsening shortness of breath. Her symptoms were accompanied by a productive cough, fatigue, fever, and left-sided pleuritic chest pain exacerbated by inspiration. The patient had been recently discharged from the hospital one month back after being treated for acute hypoxic respiratory failure secondary to bilateral pleural effusions, with a right chest tube placed during that admission to drain exudative fluid.

**Figure 1:**
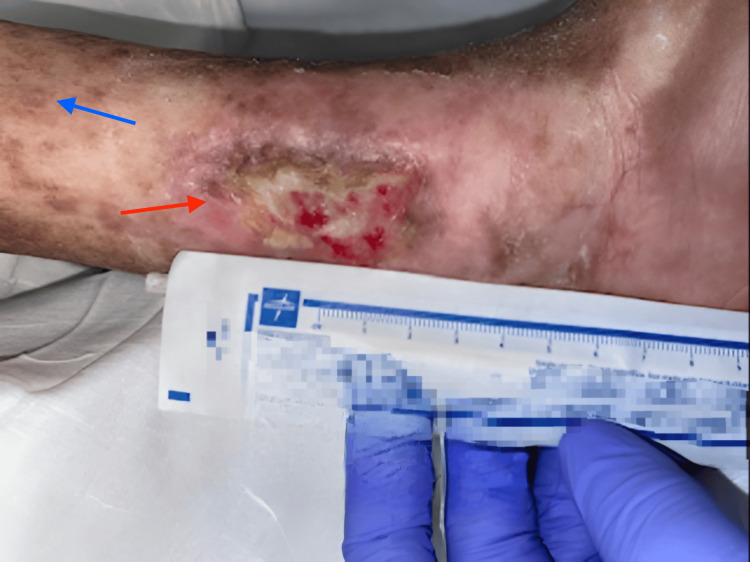
Chronic skin pigmentation (indicated by the blue arrow) and an ulcer on the left medial malleolus (indicated by the red arrow).

In the ED, her vital signs revealed a heart rate of 110 beats per minute, blood pressure of 119/81 mmHg, respiratory rate of 19 breaths per minute, and oxygen saturation of 95% on room air. She denied headaches, dizziness, abdominal pain, or changes in bowel habits. She had dry skin with chronic pigmentation and an ulcer on the left medial malleolus, as shown in Figure 1. Chest examination revealed bilaterally reduced air entry without crepitation, while cardiac, abdominal, and neurological examinations were unremarkable.

Initial lab work revealed leukocytosis with a white blood cell count of 11.6 × 109/L, mild macrocytic anemia, and an elevated C-reactive protein. The chest X-ray demonstrated bilateral pleural effusions, more prominent on the left side, similar to prior imaging. The patient met systemic inflammatory response syndrome (SIRS) criteria with tachycardia, tachypnea, and leukocytosis, raising concern for sepsis secondary to pleural effusion versus pneumonia. Despite broad-spectrum antibiotic coverage with cefepime and doxycycline, her fever and respiratory symptoms persisted. Initial lab values are tabulated in Table 1.

**Table 1:** Initial laboratory values showed mild leukocytosis and macrocytic anemia.

Parameter	Initial Lab Values	Normal Range
White Blood Cell (WBC) Count (×10^9^/L)	11.6	4.0-11.0
Red Blood Cell (RBC) Count (×10^12^/L)	2.75	4.2-5.9
Hemoglobin (HGB) (g/dL)	9.5	13.0-17.0
Hematocrit (HCT) (%)	31.7	38.3-48.6
Mean Corpuscular Volume (MCV) (fL)	106.7	80.0-100.0
Mean Corpuscular Hemoglobin (MCH) (pg)	34.5	27.0-33.0
Mean Corpuscular Hemoglobin Concentration (MCHC) (g/dL)	33.2	33.4-35.5
Red Cell Distribution Width (RDW) (%)	15.2	11.5-14.5
Platelet Count (×10^9^/L)	312	150-400
Neutrophil Count (%)	69.6	40-70
Sodium (Na) (mmol/L)	134	135-145
Potassium (K) (mmol/L)	4.0	3.5-5.1
Chloride (Cl) (mmol/L)	109	98-107
Carbon Dioxide (CO2) (mmol/L)	17	22-29
Glucose (mg/dL)	74	70-140
Blood Urea Nitrogen (BUN) (mg/dL)	19.5	6-20
Creatinine (Cr) (mg/dL)	1.4	0.6-1.2
Calcium (Ca) (mg/dL)	7.7	8.5-10.2
Anion Gap (mmol/L)	12.0	3-11
Estimated Glomerular Filtration Rate (eGFR) (mL/minute/1.73 m²)	>60	>60

Her antibiotics were escalated to piperacillin-tazobactam for broader coverage, but she continued to experience febrile episodes with a peak temperature of 102.8°F. Blood cultures, respiratory viral panels, and MRSA screening were negative. Despite initial improvements with supplemental oxygen (2 L/minute), she became more tachypneic and developed hypotension, requiring midodrine to stabilize her blood pressure.

**Figure 2:**
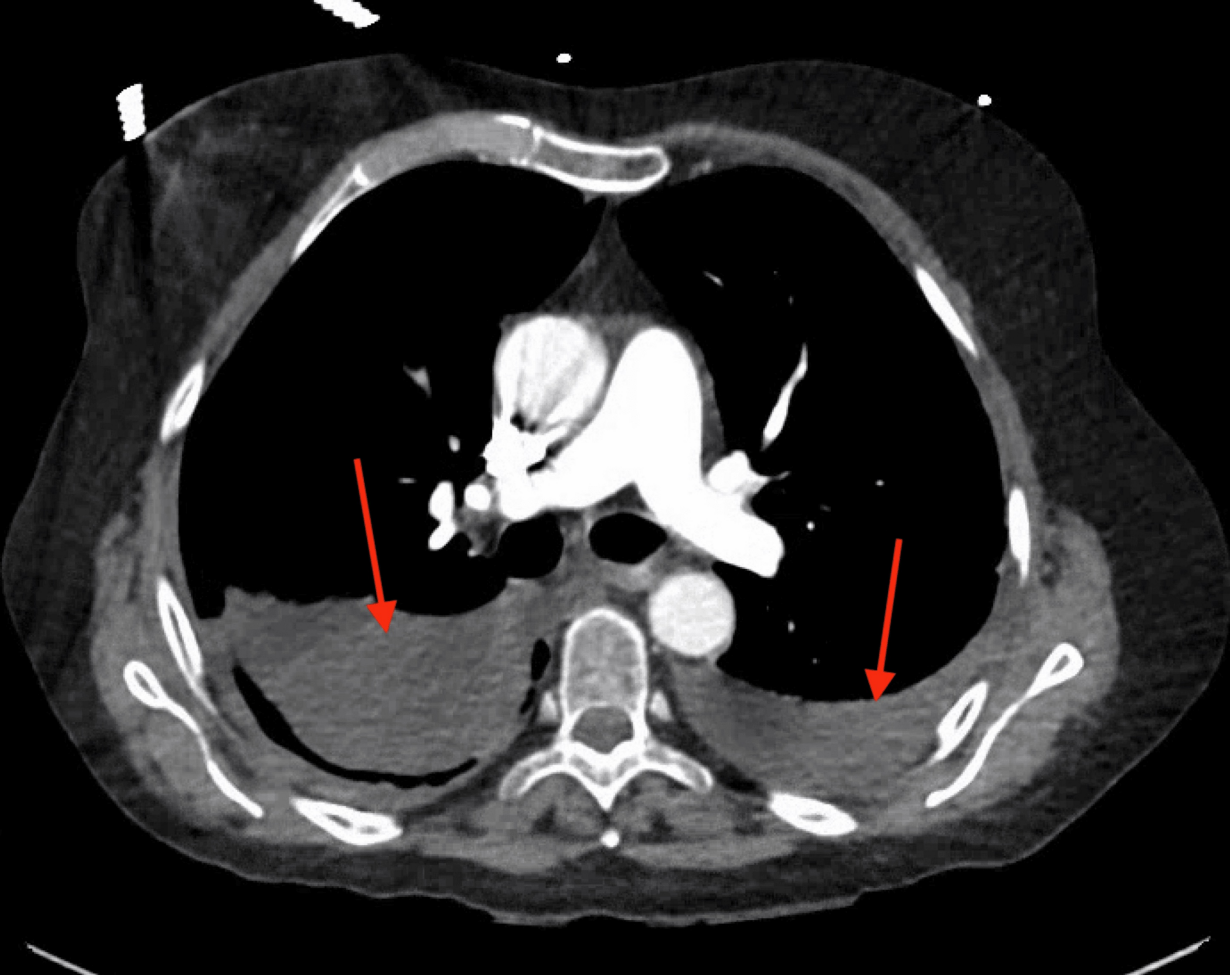
CT angiography of the chest shows bilateral pleural effusion, indicated by the red arrows.

**Figure 3:**
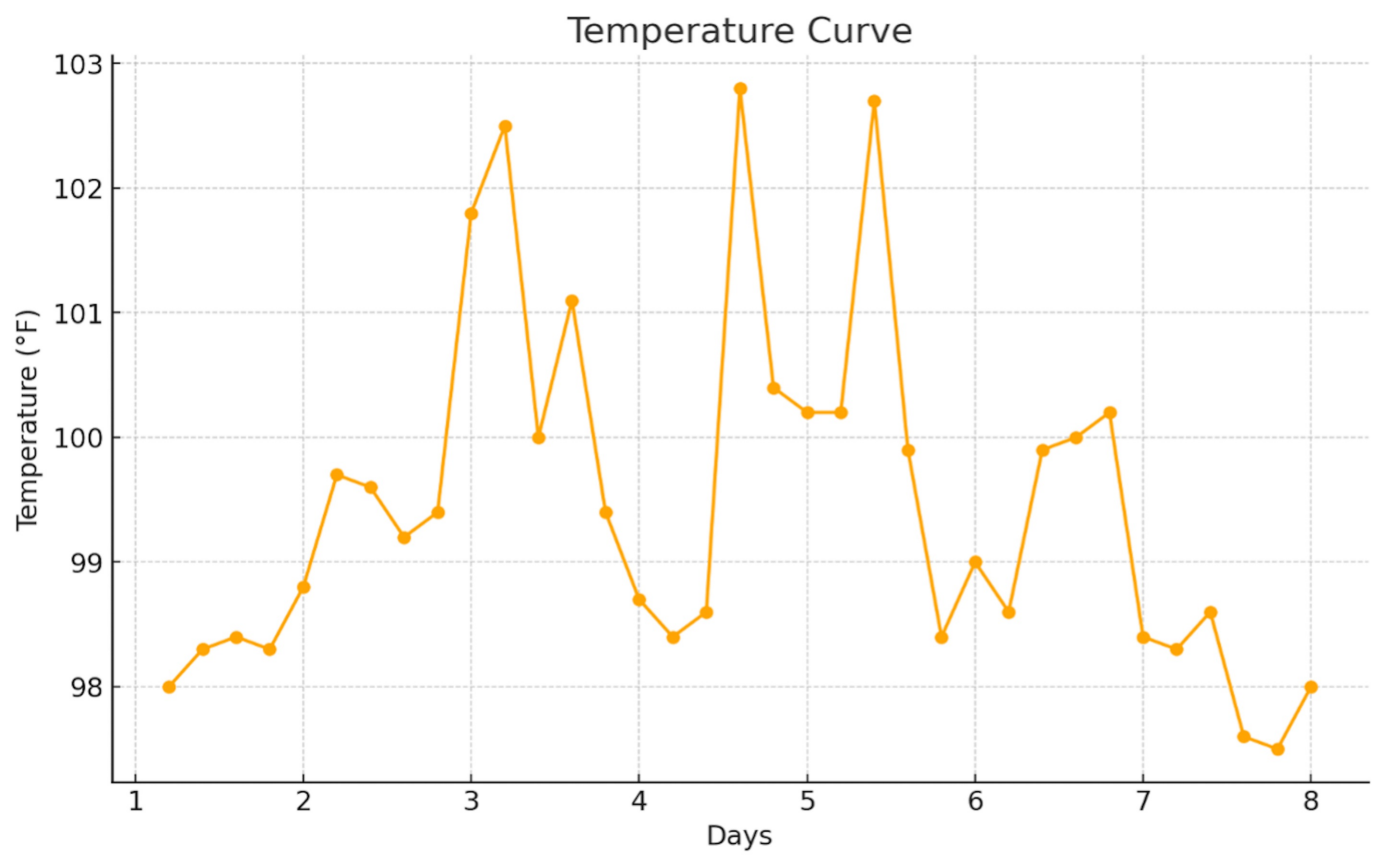
Temperature curve during hospital stay.

A computed tomography angiography (CTA) of the chest ruled out pulmonary embolism (PE), and repeat imaging showed worsening pulmonary infiltrates, as shown in Figure 2. Given the patient's history of allogeneic BMT and persistent respiratory distress, a multidisciplinary team, including hematology, oncology, and pulmonary specialists, was consulted. The possibility of cGVHD involving the lungs and skin was considered. The patient was started on oral prednisone 40 mg twice daily for presumed cGVHD. The temperature curve shown in Figure 3 displays an initial rise in temperature, which returned to baseline after the initiation of steroids.

Remarkably, her clinical condition improved rapidly within 24 hours of steroid therapy. Her oxygen requirement decreased, tachycardia resolved, and follow-up imaging demonstrated improvement in bilateral pulmonary infiltrates. The patient was medically stable, but due to her persistent oxygen needs and physical deconditioning, physical therapy recommended short-term rehabilitation. However, the patient expressed a preference to return home, where she had adequate family support. She was discharged home with supplemental oxygen, a wheelchair, and instructions for home physical therapy.

The patient was advised to follow up with her hematology, oncology, and transplant teams to manage her cGVHD and recurrent pleural effusions.

## Discussion

This case report highlights the complexities of diagnosing and managing cGVHD in a 53-year-old female patient with a history of ALL who underwent allogeneic BMT. Her clinical presentation, characterized by respiratory distress, pleuritic chest pain, and distinctive skin findings, raises important considerations regarding the diagnosis and management of post-transplant complications.

cGVHD generally develops more than 100 days after allogeneic hematopoietic cell transplantation (HSCT) as the donor immune cells mount an attack against the recipient's tissues [3]. GVHD is a significant contributor to late non-relapse complications and mortality, accounting for about 25% of deaths after allogeneic HSCT [4]. The pathophysiology of cGVHD differs from acute GVHD, primarily involving impaired immune tolerance and contributions from both autoreactive and alloreactive donor-derived T and B cells [3]. Factors, such as antigen presentation by donor cells, chronic inflammation, and variations in immune reconstitution, influenced by age and thymic function, contribute to the complexity and unpredictability of outcomes in diverse patient populations [3].

cGVHD is classified into several types based on the organs affected and the symptoms presented. Classic cGVHD involves multiple organ systems, often resembling autoimmune disorders, while limited cGVHD primarily affects the skin and tends to have a more favorable prognosis. In cGVHD, the skin is commonly involved, exhibiting a variety of morphological changes depending on the affected skin layers. Typical manifestations encompass erythema, maculopapular rashes, and itching [5]. Distinctive diagnostic features may include poikiloderma, changes resembling lichen planus, and deep sclerotic lesions [5]. Although some alterations, such as depigmentation and papulosquamous lesions, can occur, these require histopathological evaluation if they do not align with recognized diagnostic criteria.

In contrast, extensive cGVHD impacts multiple organs, such as the liver and lungs, leading to more severe complications. The pulmonary cGVHD can result in conditions like bronchiolitis obliterans (BO), significantly impairing respiratory function. Pulmonary complications are the major cause of late mortality following allogeneic HSCT [6]. These complications include restrictive or obstructive lung diseases such as BO, which is diagnostic of cGVHD and carries a poor prognosis due to its resistance to treatment [3,6]. Other associated conditions include cryptogenic organizing pneumonia (COP) and interstitial pneumonitis, which may occur with cGVHD but are less specific [6]. Additionally, ocular and oral cGVHD lead to dryness and irritation in the eyes and mouth, respectively, affecting the patient’s quality of life. Understanding these classifications is essential for effective diagnosis and treatment planning in patients with cGVHD.

cGVHD is diagnosed based on the presence of specific symptoms across eight organs, laboratory values indicative of hepatic involvement, and pulmonary function tests (PFTs). Each organ is assigned a grade from 0 to 3, and the overall severity of cGVHD is categorized as mild, moderate, or severe, depending on the number of organs affected and the severity of their involvement. Specifically, mild severity is defined by one to two involved organs with mild symptoms (excluding the lungs), moderate severity by more than three organs with mild to moderate symptoms (with the lungs being only mildly affected), and severe by more than three organs, with lung involvement classified as moderate or severe as presented in Table 2 [7]. In cases where diagnostic symptoms of cGVHD are absent, histological confirmation may be necessary, particularly for gastrointestinal, nonspecific cutaneous, hepatic, and pulmonary manifestations, to exclude toxic, infectious, or comorbid causes [7]. Evidence suggests a significant risk of misdiagnosis and inappropriate treatment if cGVHD is diagnosed solely based on clinical symptoms without supporting histological findings. Ongoing research is exploring biomarkers for cGVHD, though these require further validation before being implemented in clinical practice.

**Table 2:** Severity grading of chronic graft-versus-host disease [7].

Overall Severity	Mild	Moderate	Severe
Number of Involved Organs	1-2	>3	>3
Severity of Involved Organs	Mild (Excluding Lung)	Mild-Moderate (Lung Only Mild)	Severe (Lung Moderate or Severe)

For mild cutaneous cGVHD, topical immunosuppressive therapies like steroids, calcineurin inhibitors, or phototherapy are recommended [8]. If topical treatments are not feasible, prednisone can be given. For moderate to severe forms of cutaneous cGVHD, systemic treatment with prednisone or methylprednisolone at 1 mg/kg/day is preferred, possibly combined with a calcineurin inhibitor [8,9]. Rituximab is also being explored for first-line treatment but may increase infection risks [8,9]. Response to treatment should be assessed after eight weeks, with long-term treatment often lasting three to six months. If symptoms do not improve within eight to 12 weeks, second-line therapy should be initiated.

The European Respiratory Society (ERS) and the European Society for Blood and Marrow Transplantation (EBMT) task force recommends managing pulmonary cGVHD and BO in adults through the use of inhaled corticosteroids/long-acting beta-agonists (ICS/LABA) and a combination therapy (azithromycin and montelukast) [10]. For progressive disease, extracorporeal photopheresis (ECP) and lung transplantation are options for end-stage cases. While ruxolitinib and belumosudil require more data for BOS management, imatinib and ibrutinib show less convincing efficacy and have notable toxicity, leading to a recommendation against their use [10].

In our patient, the suspicion of cGVHD was warranted, given her history of allogeneic BMT and the development of respiratory symptoms. Notably, symptoms, such as a productive cough, fever, and tachycardia, are consistent with pulmonary involvement, which may not present with the typical triad of symptoms (involving skin, liver, and gastrointestinal systems). Additionally, the presence of chronic skin pigmentation and an ulcer on the left medial malleolus, as shown in Figure 1, are highly indicative of cutaneous GVHD. These findings underline the necessity for clinicians to recognize that GVHD can manifest in various ways, often insidiously. The patient’s clinical presentation posed significant diagnostic challenges due to overlapping symptoms associated with infection and GVHD. Her leukocytosis and elevated inflammatory markers initially suggested a potential infectious process, particularly pneumonia, which is common in immunocompromised patients. The presence of bilateral pleural effusions on imaging further complicated the diagnosis, necessitating a comprehensive evaluation of both infectious and inflammatory causes.

The chronic skin pigmentation observed in the patient is particularly noteworthy. It is highly specific for cutaneous GVHD and reflects the underlying pathophysiology of the condition. The presence of skin findings can often precede other systemic manifestations of GVHD and serves as an important diagnostic clue. In this patient, the skin findings, along with respiratory symptoms, create a compelling case for cGVHD as the underlying cause of her clinical deterioration. The initiation of corticosteroid therapy with prednisone led to a rapid improvement in the patient’s clinical status, consistent with established protocols for treating acute exacerbations of GVHD. The swift resolution of symptoms and improvement in imaging findings strongly support the diagnosis of GVHD. While corticosteroids are effective in managing GVHD, they also carry risks of immunosuppression, making patients susceptible to opportunistic infections [10]. Therefore, careful monitoring is essential after starting steroid therapy to identify any complications that may arise.

## Conclusions

In conclusion, this case report illustrates the complexity of managing patients with hematologic malignancies and the potential complications that can arise post-treatment, such as cGVHD. This case report highlights the critical understanding that not every febrile episode in post-transplant patients is due to infection alone; instead, fever can arise from a multitude of non-infectious factors, including the inflammatory response associated with GVHD. A multidisciplinary approach is essential in these cases, integrating expertise from oncology, pulmonology, and transplant medicine to ensure accurate diagnosis and optimal patient management. As our understanding of the pathophysiology of cGVHD and its manifestations evolves, ongoing research and individualized patient care remain vital in improving outcomes for those affected by this condition.
